# General synthesis of complex nanotubes by gradient electrospinning and controlled pyrolysis

**DOI:** 10.1038/ncomms8402

**Published:** 2015-06-11

**Authors:** Chaojiang Niu, Jiashen Meng, Xuanpeng Wang, Chunhua Han, Mengyu Yan, Kangning Zhao, Xiaoming Xu, Wenhao Ren, Yunlong Zhao, Lin Xu, Qingjie Zhang, Dongyuan Zhao, Liqiang Mai

**Affiliations:** 1 State Key Laboratory of Advanced Technology for Materials Synthesis and Processing, Wuhan University of Technology, Wuhan 430070, China; 2 Department of Chemistry and Chemical Biology, Harvard University, Cambridge, Massachusetts 02138, USA

## Abstract

Nanowires and nanotubes have been the focus of considerable efforts in energy storage and solar energy conversion because of their unique properties. However, owing to the limitations of synthetic methods, most inorganic nanotubes, especially for multi-element oxides and binary-metal oxides, have been rarely fabricated. Here we design a gradient electrospinning and controlled pyrolysis method to synthesize various controllable 1D nanostructures, including mesoporous nanotubes, pea-like nanotubes and continuous nanowires. The key point of this method is the gradient distribution of low-/middle-/high-molecular-weight poly(vinyl alcohol) during the electrospinning process. This simple technique is extended to various inorganic multi-element oxides, binary-metal oxides and single-metal oxides. Among them, Li_3_V_2_(PO_4_)_3_, Na_0.7_Fe_0.7_Mn_0.3_O_2_ and Co_3_O_4_ mesoporous nanotubes exhibit ultrastable electrochemical performance when used in lithium-ion batteries, sodium-ion batteries and supercapacitors, respectively. We believe that a wide range of new materials available from our composition gradient electrospinning and pyrolysis methodology may lead to further developments in research on 1D systems.

## Abstract (web summary)

Nanowires and nanotubes are ideal candidates for energy applications but inorganic multielement oxides are less well studied. Here, the authors propose a gradient-electrospinning followed by controlled-pyrolysis method to synthesize various controllable one dimensional metal oxide nanostructures.

## Graphical abstract

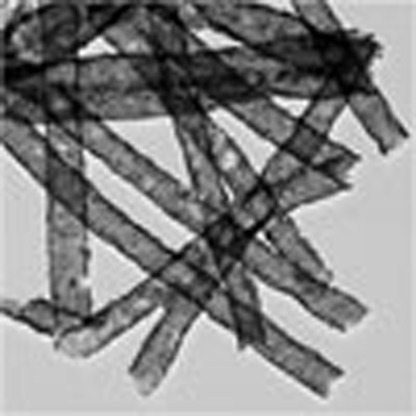

One-dimensional (1D) nanostructures, including nanowires and nanotubes, have been a focus of nanoscience and nanotechnology, due to the unique low-dimensional properties^1^,^2^,^3^,^4^,^5^,^6^. Various successful fabrication techniques, such as chemical/physical vapour deposition, hydrothermal, template based, electrochemical etching/deposition, laser ablation and electrospinning methods^7^,^8^,^9^,^10^,^11^,^12^,^13^,^14^,^15^, have been developed for specific materials. However, owing to the restriction of applicable objects of each synthetic method and the difference of crystal growth orientation of different substances^16^,^17^,^18^, 1D nanostructures are only achievable for some specific materials. Most inorganic nanotubes, especially for multi-element oxides and binary-metal oxides, have been scarcely obtained, which greatly restricts their further developments. Therefore, a universal technique is required, which can be used to fabricate nanotubes, as well as nanowires, for various inorganic materials with taking no account of the limitation of crystal orientation.

Electrospinning techniques have been studied for the fabrication of conductive polymer nanowires and a part of inorganic material nanowires^19^,^20^,^21^. This method along with different post-treatments has been applied to synthesize some interesting surface multilevel structures (branched nanowires and necklace-like nanowires) and inner multilevel structures (core/shell nanowires and multichannel microtubes)^22^,^23^,^24^,^25^,^26^. However, most of these structures have been confined to solid nanowires rather than nanotubes. Xia and co-workers have proposed a melt coaxial electrospinning method, with a coaxial spinneret, for fabricating core-shell nanowires and metal nanotubes^27^,^28^,^29^. Jiang and co-workers have developed a multifluidic compound-jet electrospinning technique for fabricating biomimic multichannel microtubes^30^. Nevertheless, it has been rarely proposed to electrospin nanotubes for different kinds of inorganic oxides, especially for multi-element oxides and binary-metal oxides, with abundant materials diversity, low cost, good repeatability and high yield, which seriously limit their further applications.

Here we design a universal gradient electrospinning followed by controlled pyrolysis methodology to synthesize various types of mesoporous nanotubes and pea-like nanotubes (Fig. 1), including multi-element oxides, binary-metal oxides and single-metal oxides. This strategy is achieved through electrospinning with one ordinary syringe needle while modulating low-, middle- and high-molecular-weight poly(vinyl alcohol) (PVA) in the precursor. In this way, different nanotubes are obtained using controllable heat treatments. The resulting mesoporous nanotubes are composed of ultrathin carbon nanotubes (∼5 nm in thickness and over 10 μm in length) and small nanoparticles (approximately 5–20 nm in diameter) on the tube walls. Pea-like nanotubes are composed of outer carbon nanotubes (∼20 nm in thickness) and nanoparticles (approximately 100–300 nm in diameter) in the nanotubes. These structures have larger specific surface area and higher ionic–electronic conductivity compared with traditional nanowires, which exhibits great potential in energy storage fields. Therefore, Li_3_V_2_(PO_4_)_3_, Na_0.7_Fe_0.7_Mn_0.3_O_2_ and Co_3_O_4_ mesoporous nanotubes were selected as electroactive materials in lithium-ion batteries, sodium-ion batteries and supercapacitors, respectively.

## Results

### Gradient electrospinning and controlled pyrolysis mechanism

**Figure 1:**
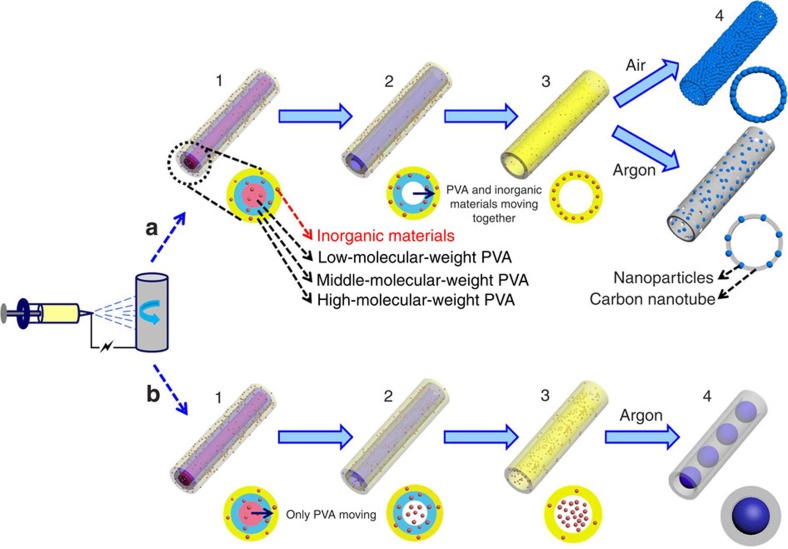
Schematics of the gradient electrospinning and controlled pyrolysis method. (**a**) Preparation process of mesoporous nanotubes. (1) After the electrospinning process, the low-, middle- and high-molecular-weight PVA tend to be distributed into three layers in the radial direction of composite nanowires. (2) As the temperature is slowly increased, the inner low-weight PVA first pyrolyses and moves towards the boundary of the low-/middle-weight PVA, carrying the inorganic materials. Then the middle-weight PVA pyrolyses and moves towards the high-weight PVA as well. (3) All of the preliminary pyrolysed PVA and inorganic materials converge together in the tube walls. (4) After sintering in air, all of the PVA pyrolyse and uniform mesoporous nanotubes are obtained, which are composed of tiny inorganic nanoparticles. On the other hand, after high-temperature sintering under argon, PVA carbonize, uniform mesoporous nanotubes are also obtained, which consists of inorganic nanoparticles and carbon nanotubes. The mesopores result from the decomposition of the inorganic materials and a part of PVA polymers. (**b**) Preparation process of pea-like nanotubes. (1) After the electrospinning process, the composite nanowires are directly and immediately placed into a furnace in air, which is preheated to and maintained at 300 °C. (2, 3) All of the PVA decompose at the same time and rapidly move towards the outer high-weight PVA layer without carrying the inorganic materials, leaving them in the centre. (4) After high-temperature sintering under argon, the outer PVA carbonize and the inner inorganic materials develop into nanoparticles, forming pea-like nanotubes.

**Figure 2:**
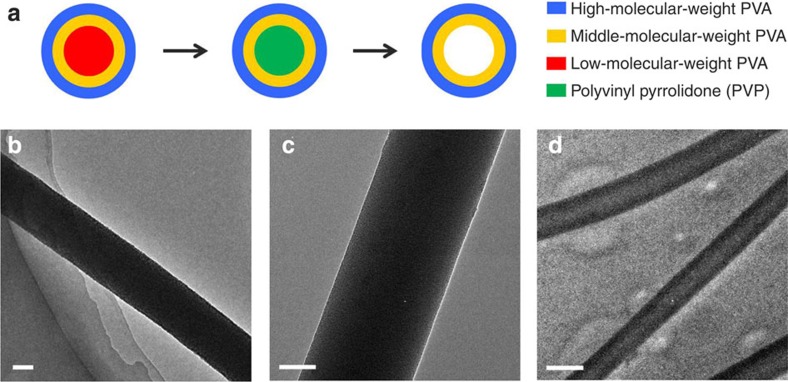
Schematic and characterization of the gradient distribution of low-/middle-/high-molecular-weight PVA. (**a**) Schematic of the process of replacing low-molecular-weight PVA by PVP, then removing PVP with trichloromethane, to prove the layered distribution of low-, middle- and high-molecular-weight PVA. (**b**) TEM image of low-, middle- and high-molecular-weight PVA composite polymer nanowire after electrospinning with a scale bar at 100 nm. (**c**) TEM image of PVP and middle-/high-molecular-weight PVA composite polymer nanowire after electrospinning with a scale bar at 50 nm. Low-molecular-weight PVA is replaced by PVP. (**d**) TEM image of middle-/high-molecular-weight PVA polymer nanotubes with a scale bar at 500 nm, after removing PVP in the inner center using CHCl_3_.

First, the viscous homogeneous precursor solution was prepared by mixing low-, middle- and high-molecular-weight PVA (in a weight ratio of 3:2:1) and different needed inorganic materials. The precursor solution was then delivered into an ordinary metallic needle at a constant flow rate and electrospun at 20 kV. Under the strong electrostatic tension force, the low-, middle- and high-molecular-weight PVA tend to be separated into three layers instead of mixing together. The reasons are as follows: at the same electrospinning conditions, the electric field (*E*), flow rate (*Q*), electric current (*I*), distance (*D*) between the injector nozzle and the receiver, and other operating characteristics of the low-, middle- and high-molecular-weight PVA are the same. According to the two equations proposed by Baumgarten and Rutledge^31^,^32^ (equations. 1, 2),


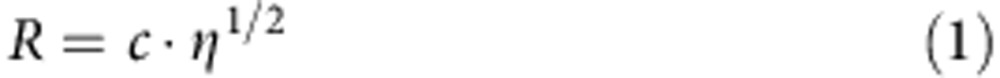



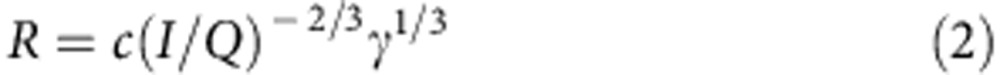


where *R* is the terminal jet radius, *η* is the viscosity, *c* is a constant, *γ* is the surface tension, *Q* is the flow rate and *I* is the electric current. The terminal jet radius (*R*) is directly proportional to the square root of the viscosity (*η*^1/2^) and to the cube root of the surface tension (*γ*^1/3^). The *η* values of the low-, middle- and high-molecular-weight PVA were measured as 0.0766, 0.5350 and 0.7685, dl g^−1^, respectively, increasing gradually. The ***γ*** values of these three PVA were tested as 40.1, 41.6 and 51.4 mN m^−1^, respectively (Supplementary Fig. 1a-c, g). Therefore, the high-weight PVA was distributed in the outer layer, the middle-weight PVA was located in the middle layer, and the low-weight PVA was concentrated in the centre in theory (Fig. 1a). To prove this important viewpoint, low-weight PVA was replaced by polyvinyl pyrrolidone (PVP), which has much smaller molecular weight, viscosity and surface tension (Fig. 2a, Supplementary Fig. 1g). After electrospinning low-, middle- and high-molecular-weight PVA composite polymer, the sample is solid nanowires, as shown in transmission electron microscope (TEM) image (Fig. 2b). After replacing the low-weight PVA by PVP, the sample is solid nanowires as well (Fig. 2c). Herein, PVP can be dissolved in trichloromethane (CHCl_3_) solution, but PVA can not be dissolved in it. Therefore, the composite polymer nanowires were soaked in the CHCl_3_ to remove PVP, getting middle-/high-molecular-weight PVA polymer nanotubes (Fig. 2d), which can clearly prove the layered distribution of low-, middle- and high-molecular-weight PVA. At the same time, the inorganic materials were homogeneously dispersed in all three layers.

After the electrospinning process, the composite nanowires were presintered in air. According to the thermogravimetric curves, the differentials of the mass loss (*M*) and temperature (*T*) (d*M*/d*T*) of the low-, middle- and high-weight PVA are −1.50, −0.95 and −0.58, respectively (Supplementary Fig. 1d,e). Consequently, the inner low-weight PVA first pyrolyzes and shrinks as the temperature is slowly increased, and moves towards the boundary of the low-/middle-weight PVA, carrying the inorganic materials simultaneously, thereby leading to the formation of nanotubes^33^. By this analogy, the middle-weight PVA then pyrolyzes and moves towards the middle-/high-weight PVA, together with the inorganic materials, thereby leading to the expansion of the inner diameter of nanotubes, as illustrated in Fig. 1a. Finally, all of the preliminarily decomposed PVA and inorganic materials converge together on the outer tubes. At last, inorganic mesoporous nanotubes can be obtained after sintering in air, which are only composed of tiny inorganic nanoparticles. Mesopores are formed via the decomposition of the inorganic materials and the partial pyrolysis of PVA. On the other hand, after high-temperature sintering under argon, all of the PVA carbonize, resulting in composite mesoporous nanotubes, which are composed of uniform inorganic nanoparticles and ultrathin carbon nanotubes.

For pea-like nanotubes, the preliminary electrospinning process of pea-like nanotubes is the same as that of the mesoporous nanotubes. After electrospinning, the composite nanowires were directly and immediately placed into the furnace, which was preheated to 300 °C in air. At this temperature, all of the low-/middle-/high-weight PVA simultaneously decompose (Supplementary Fig. 1d) and quickly move towards the outer high-weight PVA layer without carrying the inorganic materials in the radial direction (Fig. 1b). Thus, the inorganic materials are left *in situ* in the centre of the nanotubes. When the samples are annealed at high temperature under argon, the outer preliminary decomposed PVA carbonize and form carbon nanotubes, whereas the inner inorganic materials develop into nanoparticles, which are uniformly dispersed in the nanotubes. Eventually, pea-like nanotubes are obtained.

**Figure 3:**
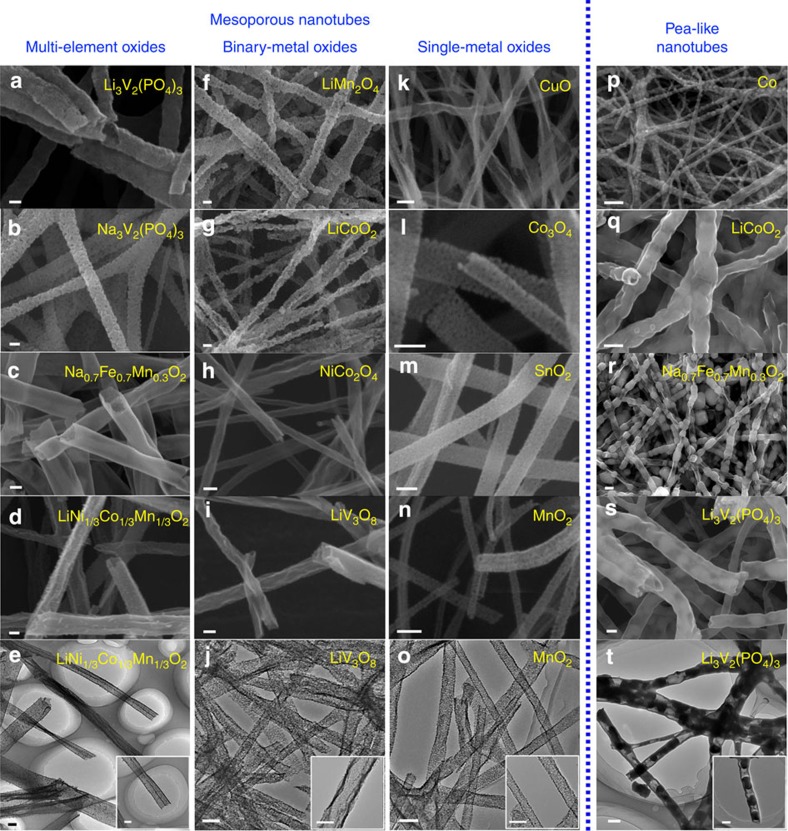
Expansion of the gradient electrospinning and controlled pyrolysis method. (**a**–**o**) SEM and TEM images of multi-element oxides (Li_3_V_2_(PO_4_)_3_, Na_3_V_2_(PO_4_)_3_, Na_0.7_Fe_0.7_Mn_0.3_O_2_ and LiNi_1/3_Co_1/3_Mn_1/3_O_2_), binary-metal oxides (LiMn_2_O_4_, LiCoO_2_, NiCo_2_O_4_ and LiV_3_O_8_) and single-metal oxides (CuO, Co_3_O_4_, SnO_2_ and MnO_2_) mesoporous nanotubes, respectively, scale bars, 100 nm. (**p**–**t**) SEM and TEM images of pea-like nanotubes (Co, LiCoO_2_, Li_3_V_2_(PO_4_)_3_ and Na_0.7_Fe_0.7_Mn_0.3_O_2_) from different species with scale bars at 200 nm. The scale bars for the inset TEM images (**e**, **j**, **o**, **t**) are 100 nm.

To confirm the mechanism of our gradient electrospinning and controlled pyrolysis method, various inorganic materials were electrospun into mesoporous nanotubes and pea-like nanotubes according to the aforementioned procedures (Fig. 3). First, multi-element oxides (Li_3_V_2_(PO_4_)_3_, Na_3_V_2_(PO_4_)_3_, Na_0.7_Fe_0.7_Mn_0.3_O_2_ and LiNi_1/3_Co_1/3_Mn_1/3_O_2_) were electrospun into uniform mesoporous nanotubes with a diameter of ∼200 nm. Then, the binary-metal oxides (LiMn_2_O_4_, LiCoO_2_, NiCo_2_O_4_ and LiV_3_O_8_) were electrospun into mesoporous nanotubes with a diameter of ∼150 nm. For single-metal oxides (CuO, Co_3_O_4_, SnO_2_ and MnO_2_), mesoporous nanotubes with a smaller diameter of ∼50 nm were fabricated. For the pea-like nanotubes, Co, LiCoO_2_, Li_3_V_2_(PO_4_)_3_ and Na_0.7_Fe_0.7_Mn_0.3_O_2_ were selected from the different species, the outer layer was carbon and the inner particles were different inorganic salts. Additional scanning electron microscope (SEM) images and corresponding X-ray diffraction patterns of each sample are presented as well (Supplementary Figs 2 and 3). The detailed processes are clearly illustrated in the Methods section. Each precursor solution was electrospun at a constant flow rate of ∼0.1 ml h^−1^ using one ordinary syringe needle (10 ml), and the corresponding production of inorganic materials was as high as ∼0.6 mmol h^−1^ and ∼900 cm^2^ of aluminium foil for each run (Supplementary Fig. 1f), which was a large yield. And these products can also be collected in a parallel array around the rotating wheel, to improve the packing density.^34^

### Electrochemical characterization

**Figure 4:**
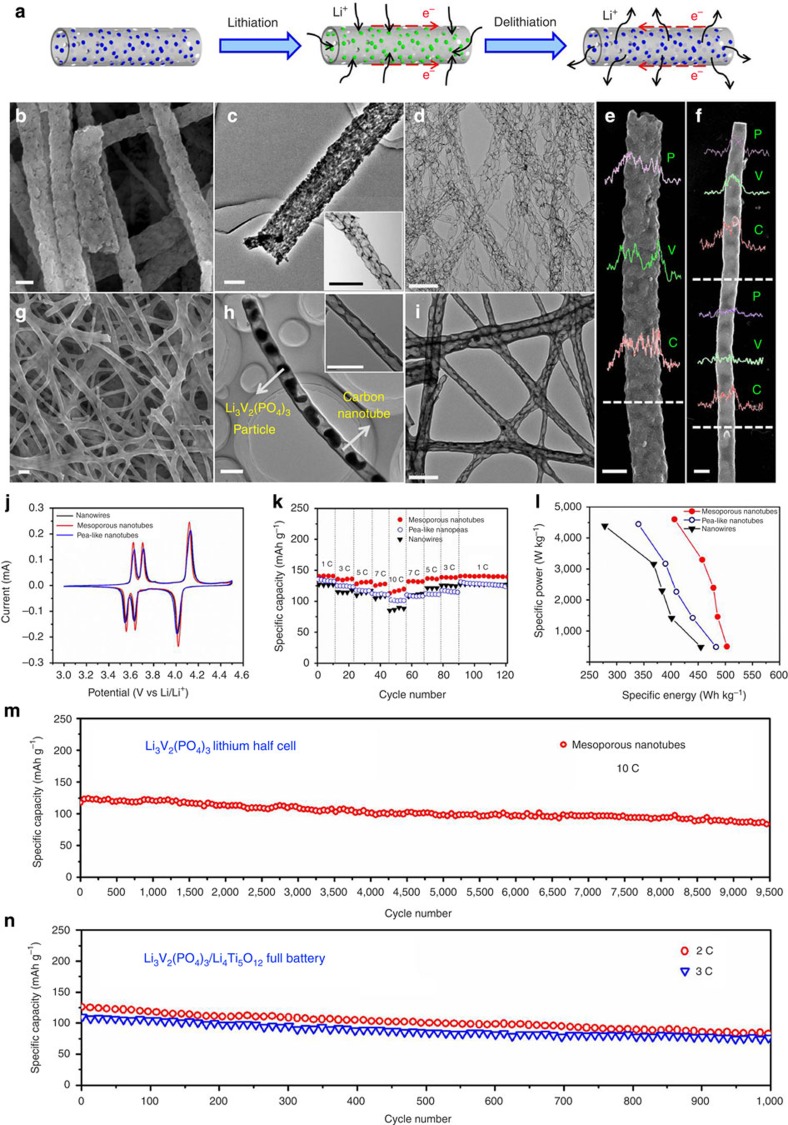
Characterization and electrochemical performance in lithium-ion batteries. (**a**) Schematic of the lithiation and delithiation processes of mesoporous nanotubes. (**b,c**), SEM and TEM images of Li_3_V_2_(PO_4_)_3_ mesoporous nanotubes with scale bar at 200 nm. (**d**) and the inset of (**c**), TEM images of ultrathin carbon nanotubes after the Li_3_V_2_(PO_4_)_3_ was removed using hydrogen fluoride with scale bar at 500 nm. (**e,f**) Energy-dispersive X-ray spectra (EDS) line scans of Li_3_V_2_(PO_4_)_3_ mesoporous nanotubes (**e**) and pea-like nanotubes (**f**) with scale bar at 200 nm, respectively. (**g,h**) SEM and TEM images of Li_3_V_2_(PO_4_)_3_ pea-like nanotubes with scale bar at 500 nm. (**i**) and the inset of (**h**) TEM images of carbon nanotubes after removing Li_3_V_2_(PO_4_)_3_ with hydrogen fluoride with scale bar at 500 nm. (**j**) Cyclic voltammograms (CV) of the half cells collected at a sweep rate of 0.1 mV s^−1^ in the potential ranging from 3 to 4.5 V versus Li/Li^+^. (**k**,**l**) Rate performance and the corresponding Ragone plots of these three cathodes measured at the rates of 1, 3, 5, 7 and 10 C, respectively. (**m**) Long cycling performance of Li_3_V_2_(PO_4_)_3_ mesoporous nanotubes measured at 10 C for a large number of 9,500 cycles in a lithium half cell. (**n**) Cycling performance of Li_3_V_2_(PO_4_)_3_/Li_4_Ti_5_O_12_ lithium-ion full batteries measured at 2 and 3 C for 1,000 cycles (1 C equals to 133 mA g^−1^).

In energy-storage fields, most electrodes reported previously for batteries and supercapacitors suffer problems associated with a low conductivity, a small electrolyte/electrode surface area and self-aggregation during the charge/discharge process, leading to unsatisfactory performance, which greatly limits their applications^35^,^36^,^37^,^38^,^39^,^40^,^41^,^42^,^43^,^44^,^45^. One-dimensional nanomaterials have widely been investigated and applied in energy storage fields due to its unique low-dimensional properties. Remarkably, our complex nanotubes, especially mesoporous nanotubes, have the characteristics of large surface area, excellent stability and continuous carbon nanotubes with high conductivity and so on, which is expected to effectively improve the electrochemical performance of electrodes (Fig. 4). Therefore, to confirm it, Li_3_V_2_(PO_4_)_3_, Na_0.7_Fe_0.7_Mn_0.3_O_2_ and Co_3_O_4_ mesoporous nanotubes were selected and measured as typical examples of electroactive materials in lithium-ion batteries, sodium-ion batteries and supercapacitors, respectively.

First, the Li_3_V_2_(PO_4_)_3_ mesoporous nanotubes were further characterized, measured and compared with the Li_3_V_2_(PO_4_)_3_ pea-like nanotubes and solid nanowires (Supplementary Figs. 4-7). The Li_3_V_2_(PO_4_)_3_ mesoporous nanotubes consist of ultrathin, continuous, mesoporous carbon nanotubes (∼200 nm in diameter) and Li_3_V_2_(PO_4_)_3_ nanoparticles (approximately 20–50 nm, uniformly dispersed on the tubes) (Fig. 4b–d), which is clearly confirmed by removal of the Li_3_V_2_(PO_4_)_3_ with hydrogen fluoride. Moreover, the mesopore size can also be tuned by modulating the sintering temperature (Supplementary Fig. 5). In contrast, the Li_3_V_2_(PO_4_)_3_ pea-like nanotubes are composed of outer thick carbon nanotubes (∼200 nm in diameter and ∼20 nm in wall thickness) and Li_3_V_2_(PO_4_)_3_ nanoparticles in the nanotubes (∼200 nm in diameter), which uniformly separate from each other (Fig. 4g–i). The EDS line scans demonstrate the homogeneous distribution of V, P and C in mesoporous nanotubes, and display the difference between the hollow and solid parts in pea-like nanotubes (Fig. 4e,f). The carbon mass content of Li_3_V_2_(PO_4_)_3_ mesoporous nanotubes is only 7%, which is much smaller compared with pea-like nanotubes (17%) and nanowires (12%; Supplementary Fig. 4). Li_3_V_2_(PO_4_)_3_ mesoporous nanotubes exhibit higher capacity and better rate recovery (100%) than pea-like nanotubes (94%) and nanowires (98%) when tested at various rates ranging from 1, 3, 5, 7 to 10 C, demonstrating prominent rate performance (Fig. 4j–l). Particularly, Li_3_V_2_(PO_4_)_3_ mesoporous nanotubes can operate stably for as long as 9,500 cycles at the high rate of 10 C. The 9,500th discharge capacity is 86 mAh g^−1^, which corresponds to a capacity retention of 80% and to a capacity fading of 0.0024% per cycle (Fig. 4m). However, the capacity retentions of Li_3_V_2_(PO_4_)_3_ pea-like nanotubes and solid nanowires are only 71% and 68%, respectively, after being cycled only 1,100 times at 10 C (Supplementary Fig. 6a). Then further electrochemical measurements of Li_3_V_2_(PO_4_)_3_ mesoporous nanotubes were implemented. When Li_3_V_2_(PO_4_)_3_ mesoporous nanotubes were measured at different temperatures of -20, 20 and 60 °C at a rate of 5 C, the corresponding initial capacities were 103, 131 and 136 mAh g^−1^, respectively (Supplementary Fig. 6c,d). And the coulombic efficiency of Li_3_V_2_(PO_4_)_3_ mesoporous nanotubes can be kept on ∼100% when tested at 1 C (133 mAh g^−1^) for 500 cycles (Supplementary Fig. 6e). In the meantime, we assembled Li_3_V_2_(PO_4_)_3_/Li_4_Ti_5_O_12_ lithium-ion full batteries. Li_4_Ti_5_O_12_ was selected as the anode because of its stable charge/discharge plateau (Supplementary Fig. 7a–c). This full battery delivers capacities of 118, 93, 80 and 74 mAh g^−1^ at the rates of 2, 3, 4 and 5 C, respectively, and recovers 96% of its initial capacity when the rate is decreased back to 2 C (Supplementary Fig. 7d). Notably, the Li_3_V_2_(PO_4_)_3_/Li_4_Ti_5_O_12_ battery is stably charged/discharged for over 1,000 cycles at rates of 2 and 3 C, with a capacity retention of 73% and 75%, respectively (Fig. 4n), demonstrating an excellent cycling performance and great commercial potential.

The superior performance is explained as follows: (1) Li_3_V_2_(PO_4_)_3_ mesoporous nanotubes contain ultrathin continuous carbon nanotubes. The charge transfer resistance (Rct) of Li_3_V_2_(PO_4_)_3_ mesoporous nanotubes is measured as 56 Ω, much smaller than that of nanowires (115 Ω), which can obviously improve the electronic conductivity^36^ (Supplementary Fig. 6b). (2) Li_3_V_2_(PO_4_)_3_ mesoporous nanotubes exhibit the largest specific surface area (115 m^2^ g^−1^) among mesoporous nanotubes, pea-like nanotubes (33 m^2^ g^−1^) and nanowires (15 m^2^ g^−1^), which is beneficial to increase the electrode–electrolyte contact area and the active sites^39^,^40^ (Supplementary Fig. 4). (3) These nanotubes have abundant mesopores on the tube walls, which can effectively buffer the stress induced during the charge/discharge process, and greatly improve the structural stability^40^ (Fig. 4a).

**Figure 5:**
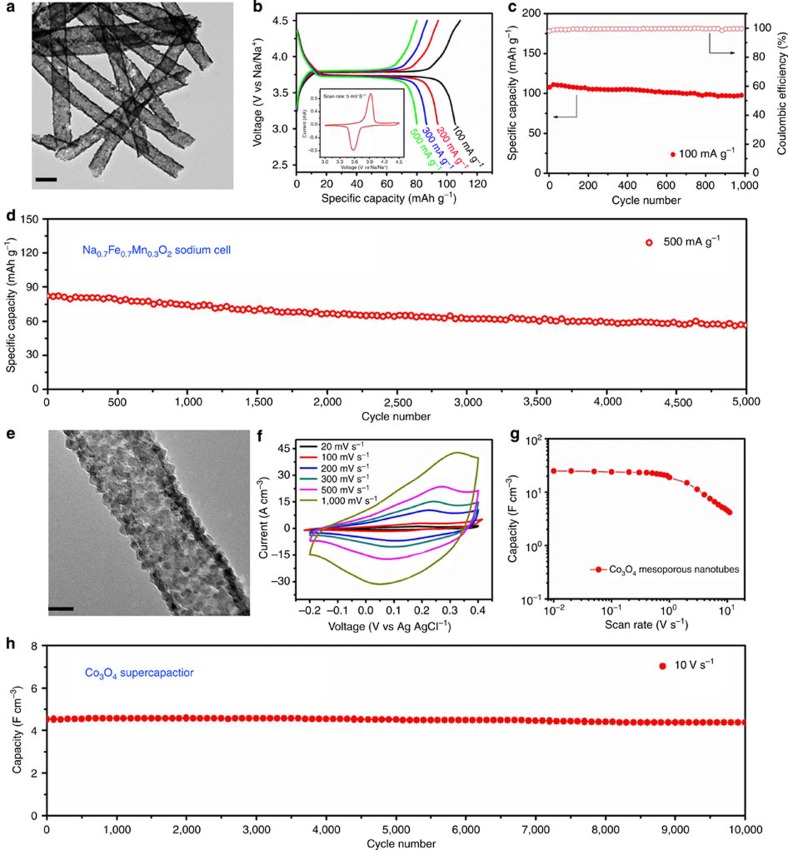
Characterization and electrochemical performance in sodium-ion batteries and supercapacitors. (**a**) TEM image of the Na_0.7_Fe_0.7_Mn_0.3_O_2_ mesoporous nanotubes with a scale at 200 nm. (**b**) Charge–discharge curves of Na_0.7_Fe_0.7_Mn_0.3_O_2_ measured at 100, 200, 300 and 500 mA g^−1^, respectively. The inset is the CV collected at a scan rate of 5 mV s^−1^ in the potential range 3.0–4.5 V. (**c,d**) Cycling performance of Na_0.7_Fe_0.7_Mn_0.3_O_2_ mesoporous nanotubes tested for 1,000 cycles at 100 mA g^−1^ and for 5,000 cycles at 500 mA g^−1^ (**e**) TEM image of Co_3_O_4_ mesoporous nanotubes with scale bar at 20 nm. (**f**) CV curves obtained at different scan rates from 20, 100, 300, 500 to 1,000 mV s^−1^, respectively. (**g**) Stack capacitance of Co_3_O_4_ mesoporous nanotubes versus scan rate. (**h**) Long cycling performance of Co_3_O_4_ mesoporous nanotubes tested for 10,000 times at a high rate of 10 V s^−1^.

The Na_0.7_Fe_0.7_Mn_0.3_O_2_ mesoporous nanotubes are also composed of ultrathin carbon nanotubes (∼200 nm in diameter) and Na_0.7_Fe_0.7_Mn_0.3_O_2_ nanoparticles (∼10 nm) on the tubes (Fig. 5a). And the X-ray diffraction pattern, ICP measurement and high-resolution TEM demonstrate the rhombohedral crystal structure of Na_0.7_Fe_0.7_Mn_0.3_O_2_ (Supplementary Fig. 8). Stable voltage plateaus are observed when measured at the current densities of 100, 200, 300 and 500 mA g^−1^, respectively, corresponding to one pair of well-defined anodic (3.9 V) and cathodic (3.6 V) peaks (Fig. 5b). When Na_0.7_Fe_0.7_Mn_0.3_O_2_ mesoporous nanotubes are tested at a low density of 100 mA g^−1^ in the potential range 3–4.5 V, 90% of the initial capacity (109 mAh g^−1^) is retained after 1,000 cycles (Fig. 5c). When measured at a high current density of 500 mA g^−1^, 70% of the initial capacity (82 mAh g^−1^) is maintained after cycling as long as 5,000 times, corresponding to a capacity fading of 0.0071% per cycle (Fig. 5d). Compared with the conventional Na_0.7_Fe_0.7_Mn_0.3_O_2_ nanoparticles, which were synthesized as a control sample, our Na_0.7_Fe_0.7_Mn_0.3_O_2_ mesoporous nanotubes exhibit much higher specific capacity and better cycling performance (Supplementary Figs 8 and 9). Another comparison with those previously reported results for sodium-ion batteries reveals that our Na_0.7_Fe_0.7_Mn_0.3_O_2_ mesoporous nanotubes demonstrate superior electrochemical performance (Supplementary Fig. 8m).

The Co_3_O_4_ mesoporous nanotubes are very uniform with a diameter of ∼50 nm, and only contain ∼5 nm nanoparticles on the tubes (Fig. 5e, Supplementary Fig. 10). A micro-supercapacitor device based on these nanotubes was fabricated on a silicon wafer (The fabrication process of micro-supercapacitor device is clearly illustrated in Supplementary Fig. 10). These cyclic voltammograms reveal the exceptionally enhanced electrochemical performance of this material (Fig. 5f). 25.0, 24.0 and 18.9 F cm^−3^ for stack capacitance are obtained at the scan rates of 0.01, 0.1 and 1 V s^−1^, respectively (Fig. 5g). When tested at a high rate of 10 V s^−1^, it delivers a capacity of 4.5 F cm^−3^, and 98% of the initial capacity is maintained after cycling as long as 10,000 times (Fig. 5h). All these unveil that Co_3_O_4_ mesoporous nanotubes demonstrate excellent electrochemical performance with respect to both high rate and long life, showing great application potential.

## Discussion

In summary, our findings clearly indicate that the feasible gradient-electrospinning and controlled-pyrolysis methodology, with extensive material diversity, low cost, good repeatability and high yield, provides an efficient strategy to obtain controllable nanotubes for various inorganic materials, which can break through the limitation of the crystal growth orientation of each sample. On the basis of the formation mechanism, we successfully applied this technique to synthesize various multi-element oxides (Li_3_V_2_(PO_4_)_3_, Na_3_V_2_(PO_4_)_3_, Na_0.7_Fe_0.7_Mn_0.3_O_2_ and LiNi_1/3_Co_1/3_Mn_1/3_O_2_), binary-metal oxides (LiMn_2_O_4_, LiCoO_2_, NiCo_2_O_4_ and LiV_3_O_8_) and single-metal oxides mesoporous nanotubes (CuO, Co_3_O_4_, SnO_2_ and MnO_2_). Therefore, we believe this technique could lead to rapid advancements in the development of 1D nanostructures. In addition, these novel mesoporous and pea-like nanotubes, which exhibit excellent electrochemical performance in lithium-ion batteries, sodium-ion batteries and supercapacitors, owing to their large surface area, high conductivity and robust structural stability, will have great potentials in not only the energy storage field, but also in numerous other frontiers.

## Methods

### Electrospinning Li_3_V_2_(PO_4_)_3_ mesoporous nanotubes

First, the uniform precursor solution for electrospinning was prepared with low-molecular-weight (98–99% hydrolysed), middle-molecular-weight (86–89% hydrolysed) and high-molecular-weight (98–99% hydrolysed) PVA in a weight ratio of 3:2:1 in 20 ml of deionized water. (This same uniform precursor solution was used to prepare the following samples.) LiOH·H_2_O, NH_4_VO_3_ and NH_4_H_2_PO_4_ were also added in a molar ratio of 3:2:3. After the mixture was stirred at 50 °C for 5 h, a viscous, uniform, transparent yellow precursor solution was obtained. The concentration of the precursor solution was ∼9.5 wt%, the average viscosity was measured as ∼0.53 dl g^−1^. The relative humidity was maintained at a constant of ∼40% RH, and the temperature was maintained at ∼20 °C. The precursor solution was subsequently electrospun at a constant flow rate of∼1 ml h^−1^ and at a high voltage of 20 kV (electrospinning equipment: SS-2534H from UCALERY Co., Beijing, China). The distance between the injector nozzle and the receiver was 15 cm. The composite nanowires were collected on revolving aluminium foil. After drying at 80 °C for 12 h, the composite nanowires were presintered at 300 °C (10 °C min^−1^) in air for 3 h. The sample was then annealed at 800 °C (5 °C min^−1^) under an argon atmosphere for 6 h to fully carbonize the decomposed PVA. Finally, uniform Li_3_V_2_(PO_4_)_3_ mesoporous nanotubes were obtained with a 7% carbon content (Supplementary Fig. 4).

### Electrospinning Na_3_V_2_(PO_4_)_3_ mesoporous nanotubes

NH_4_VO_3_ and NaH_2_PO_4_·2H_2_O were added in a molar ratio of 2:3 to the prepared PVA precursor. Then the precursor was electrospun at a high voltage of 20 kV. After the electrospinning process, the composite nanowires were sintered at 300 °C (10 °C min^−1^) in air for 5 h and then annealed at 800 °C (5 °C min^−1^) in an argon atmosphere for 5 h.

### Electrospinning Na_0.7_Fe_0.7_Mn_0.3_O_2_ mesoporous nanotubes

NaNO_3_, Fe(NO_3_)_3_·9H_2_O, and Mn(CH_3_COO)_2_·4H_2_O were added in a molar ratio of 7:7:3 to the prepared PVA precursor. Then the precursor was electrospun at a high voltage of 20 kV. After the electrospinning process, the composite nanowires were first sintered at 300 °C (10 °C min^−1^) in air for 5 h and then annealed at 700 °C (5 °C min^−1^) under argon for 8 h.

### Electrospinning LiNi_1/3_Co_1/3_Mn_1/3_O_2_ mesoporous nanotubes

CH_3_COOLi·2H_2_O, Ni(CH_3_COO)_2_·4H_2_O, Mn(CH_3_COO)_2_·4H_2_O and Co(CH_3_COO)_2_·4H_2_O were added in a molar ratio of 3:1:1:1. Then the precursor was electrospun at a high voltage of 20 kV. After the electrospinning process, the composite nanowires were annealed at 700 °C (5 °C min^−1^) in air for 5 h.

### Electrospinning LiMn_2_O_4_ mesoporous nanotubes

CH_3_COOLi·2H_2_O and Mn(CH_3_COO)_2_·4H_2_O were added in a molar ratio of 1:2 to the prepared PVA precursor. The precursor was electrospun at a high voltage of 18 kV. After the electrospinning process, the composite nanowires were annealed at 700 °C (5 °C min^−1^) in air for 5 h.

### Electrospinning LiCoO_2_ mesoporous nanotubes

CH_3_COOLi·2H_2_O and Co(CH_3_COO)_2_·4H_2_O were added in a molar ratio of 1:1 to the prepared PVA precursor. The precursor was electrospun at a high voltage of 18 kV. After the electrospinning process, the composite nanowires were sintered at 500 °C (10 °C min^−1^) in air for 5 h.

### Electrospinning NiCo_2_O_4_ mesoporous nanotubes

Ni(CH_3_COO)_2_·4H_2_O and Co(CH_3_COO)_2_·4H_2_O were added in a molar ratio of 1:2 to the prepared PVA precursor. The precursor was electrospun at a high voltage of 18 kV. After the electrospinning process, the composite nanowires were sintered at 400 °C (10 °C min^−1^) in air for 5 h.

### Electrospinning LiV_3_O_8_ mesoporous nanotubes

LiOH·H_2_O and NH_4_VO_3_ were added in a molar ratio of 1:3. The precursor was electrospun at a high voltage of 18 kV. After the electrospinning process, the composite nanowires were sintered at 450 °C (10 °C min^−1^) in air for 5 h.

### Electrospinning CuO mesoporous nanotubes

A moderate amount of Cu(NO_3_)_2_ was added to the prepared PVA precursor. The precursor was electrospun at a high voltage of 16 kV. After the electrospinning process, the composite nanowires were sintered at 350 °C (10 °C min^−1^) in air for 5 h.

### Electrospinning Co_3_O_4_ mesoporous nanotubes

A moderate amount of Co(CH_3_COO)_2_·4H_2_O was added to the prepared PVA precursor. The precursor was electrospun at a high voltage of 16 kV. After electrospinning, the composite nanowires were sintered at 350 °C (10 °C min^−1^) in air for 5 h. The carbon from PVA was removed via the sintering process.

### Electrospinning MnO_2_ mesoporous nanotubes

A moderate amount of Mn(CH_3_COO)_2_·4H_2_O was added to the prepared PVA precursor. Then the precursor was electrospun at a high voltage of 16 kV. After the electrospinning process, the composite nanowires were sintered at 350 °C (10 °C min^−1^) in air for 5 h.

### Electrospinning SnO_2_ mesoporous nanotubes

A moderate amount of SnCl_4_ was added to the prepared PVA precursor. The precursor was electrospun at a high voltage of 16 kV. After the electrospinning process, the composite nanowires were sintered at 500 °C (10 °C min^−1^) in air for 5 h.

### Electrospinning Li_3_V_2_(PO_4_)_3_ pea-like nanotubes

The preliminary electrospinning process was the same as that used to prepare the mesoporous nanotubes. After drying at 80 °C for 12 h, the composite nanowires were directly and immediately heated for 1.5 h in a furnace preheated to 300 °C. After the composite nanowires were annealed at 800 °C (5 °C min^−1^) under an argon atmosphere for 6 h, uniform pea-like Li_3_V_2_(PO_4_)_3_ nanotubes with 19% carbon content were obtained (Supplementary Fig. 4).

### Electrospinning Co pea-like nanotubes

An amount of Co(CH_3_COO)_2_·4H_2_O was added to the prepared PVA precursor. Then the precursor was electrospun at a high voltage of 16 kV. After the electrospinning process, the composite nanowires were presintered in a muffle furnace at 300 °C for 1.5 h and then annealed at 600 °C under argon for 5 h.

### Electrospinning LiCoO_2_ pea-like nanotubes

CH_3_COOLi·2H_2_O and Co(CH_3_COO)_2_·4H_2_O were added in a molar ratio of 1:1 to the prepared PVA precursor. Then the precursor was electrospun at a high voltage of 18 kV. After the electrospinning process, the composite nanowires were presintered in a muffle furnace at 300 °C for 1.5 h then annealed at 600 °C (5 °C min^−1^) under argon for 5 h.

### Electrospinning Na_0.7_Fe_0.7_Mn_0.3_O_2_ pea-like nanotubes

NaNO_3_, Fe(NO_3_)_3_·9H_2_O, and Mn(CH_3_COO)_2_·4H_2_O were added in a molar ratio of 7:7:3. Then the precursor was electrospun at a high voltage of 20 kV. After the electrospinning process, the composite nanowires were presintered in a muffle furnace at 300 °C for 1.5 h and then annealed at 700 °C (5 °C min^−1^) under argon for 8 h.

### Morphology and structure characterizations

The crystallographic information of the final products was measured using a Bruker D8 Advance X-ray diffractometer equipped with a Cu Kα radiation source; the samples were scanned over the 2*θ* range from 10° to 80° at room temperature. SEM images were collected using a JEOL-7100F SEM, and TEM images were collected using a JEM-2100F TEM. The BET surface area was calculated from nitrogen adsorption isotherms measured at 77 K using a Tristar-3020 instrument. Energy-dispersive X-ray spectra were recorded using an Oxford IE250 system. thermogravimetric–DSC analyses were conducted using a STA-449C. X-ray photoelectron spectroscopy analysis was conducted on a VG Multilab 2000. Raman spectra were obtained using a Renishaw INVIA micro-Raman spectroscopy system. The surface tension was tested by an automatic surface tensiometer (CC2L202) from 2 to 200 N m^−1^.

### Preparation of Li_3_V_2_(PO_4_)_3_ lithium half cells

The 2016 coin cells were assembled in a glovebox filled with pure argon gas. Lithium foil was used as the anode, and a solution of LiPF_6_ (1 M) in EC/DEC (1:1 vol/vol) was used as the electrolyte. The cathode was composed of a ground mixture of 70% Li_3_V_2_(PO_4_)_3_ active material, 20% acetylene black and 10% poly(tetrafluoroethylene). After coating onto aluminium foil, the cathode was cut into round slice with ∼0.36 cm^2^ in area and ∼0.1 mm in thickness. And the loading rate of Li_3_V_2_(PO_4_)_3_ active material was ∼2 mg for one 2016 coin cell.

### Preparation of Li_3_V_2_(PO_4_)_3_/Li_4_Ti_5_O_12_ full batteries

The Li_3_V_2_(PO_4_)_3_/Li_4_Ti_5_O_12_ battery was also assembled in a glovebox filled with pure argon gas. Li_3_V_2_(PO_4_)_3_ was coated onto aluminium foil as the cathode, and Li_4_Ti_5_O_12_ was coated onto copper foil as the anode.

### Preparation of Na_0.7_Fe_0.7_Mn_0.3_O_2_ sodium batteries

The 2016 coin cells were assembled in a glovebox filled with pure argon gas. Sodium foil was used as the anode, and 1 M NaClO_4_ in a mixture of ethylene carbonate/dimethyl carbonate (1:2 in weight) with trace propylene carbonate (electrolyte additive) was used as the electrolyte. The prepared cathodes contained 70% Na_0.7_Fe_0.7_Mn_0.3_O_2_ active material, 20% acetylene black and 10% polyvinylidene difluoride (using *N*-methylpyrrolidone as the solvent). After coating onto aluminium foil, the cathode was cut into round slice with ∼0.36 cm^2^ in area and ∼0.1 mm in thickness, and the loading rate of Na_0.7_Fe_0.7_Mn_0.3_O_2_ was ∼2.5 mg for one 2016 coin cell.

### Preparation of Co_3_O_4_ supercapacitors

Electrochemical measurements were performed within the potential window of −0.2 to 0.4 V using an Autolab potentiostat/galvanostat (Autolab PGSTAT 302 N) in a three-electrode configuration with 1 M NaOH aqueous solution as the electrolyte; the measurements were performed at room temperature. A 1-cm^2^ piece of silicon wafer on which the Co_3_O_4_ mesoporous nanotubes were deposited was directly used as the working electrode (Supplementary Fig. 10). The reference and counter electrodes were an Ag/KCl electrode and a platinum plate, respectively.

### Electrochemical measurements

Galvanostatic charge/discharge measurements were performed using a multichannel battery testing system (LAND CT2001A). Cyclic voltammograms and electrochemical impedance spectra were collected at room temperature using an Autolab potentiostat/galvanostat.

## Additional information

**How to cite this article:** Niu, C. *et al*. General synthesis of complex nanotubes by gradient electrospinning and controlled pyrolysis. *Nat. Commun.* 6:7402 doi: 10.1038/ncomms8402 (2015).

## Supplementary Material

Supplementary Information Supplementary Figures 1-10.
