# Supplementary material for: General synthesis of complex nanotubes by gradient electrospinning and controlled pyrolysis
Source: Nat Commun. 2015 Jun 11;6:7402. doi: 10.1038/ncomms8402 (PMC4490406; doi:10.1038/ncomms8402)
Supplement: Supplementary Information — Supplementary Figures 1-10. [file ncomms8402-s1.pdf]

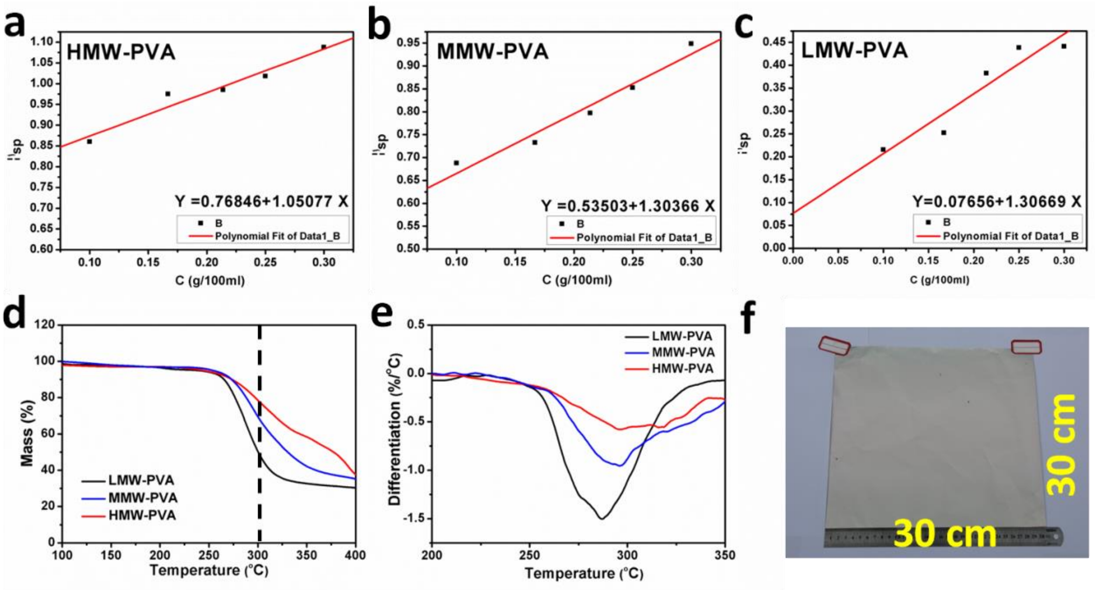

g

	25℃, K=0.0002, α=0.76, [η]=K*M^α		
	**[η] (dL/g)**	**M$_η$**	**γ (mN/m)**
**HMW-PVA**	0.76846	52067	51.4
**MMW-PVA**	0.53503	32334	41.6
**LMW-PVA**	0.07656	2504	40.1
**PVP(K13~18)**	0.01350	323	30.0

Supplementary Figure 1 | a-c, The viscosity fitting curves of high-molecular-weight poly(vinyl alcohol) (HMW-PVA) (**a**), middle-molecular-weight poly(vinyl alcohol) (MMW-PVA) (**b**) and low-molecular-weight poly(vinyl alcohol) (LMW-PVA) (**c**). **d**, TG curves of these three samples in air. **e**, The differential of the mass loss and temperature. **f**, Photograph of the aluminum foil after electrospinning for 8 h with one ordinary syringe needle. The area is ~900 cm^2. **g**, The viscosity-averaged molecular weights of HMW-PVA, MMW-PVA, LMW-PVA and PVP (K13~18).

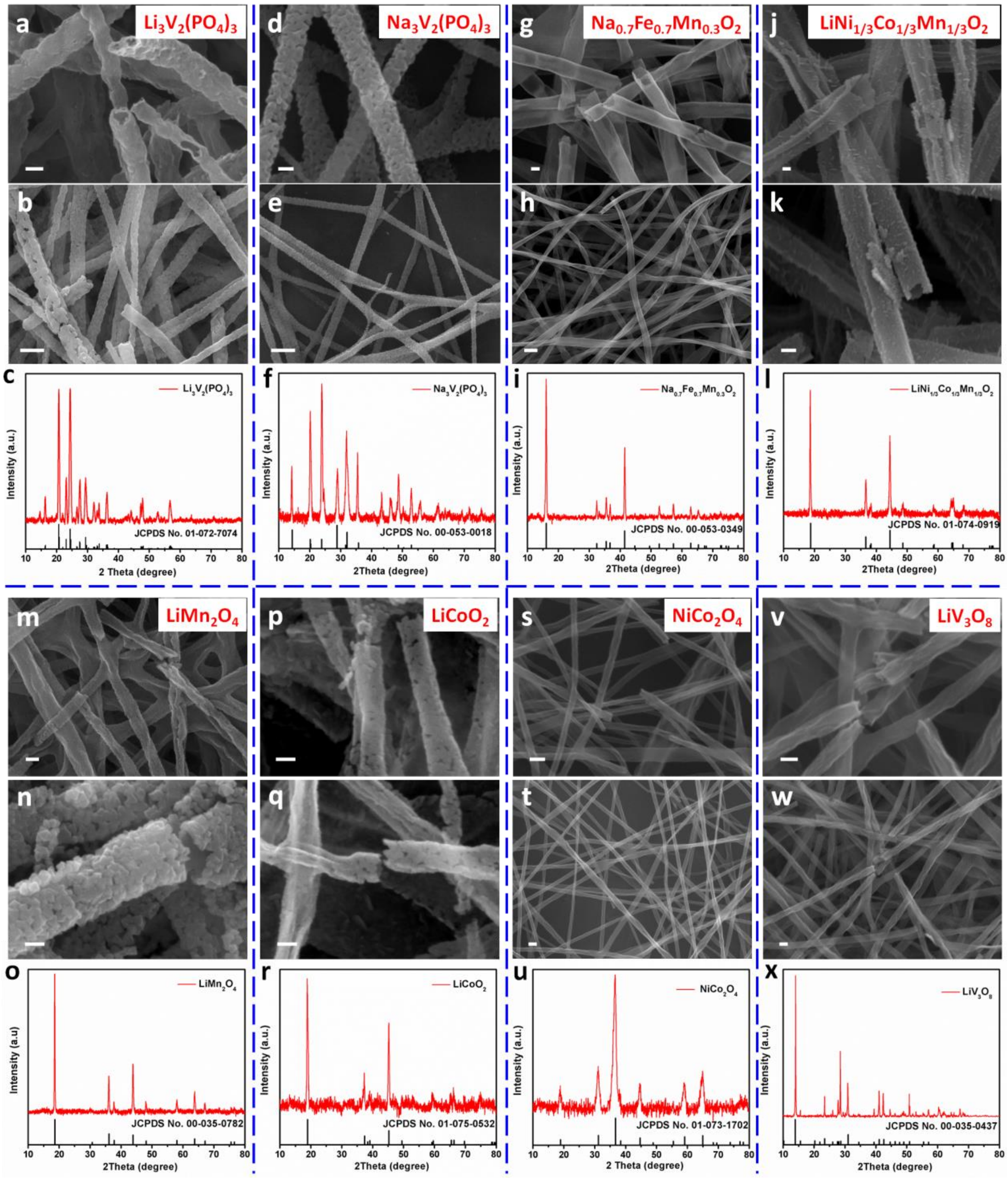

Supplementary Figure 2 | SEM images and XRD patterns of multi-element oxides, $Li_3V_2(PO_4)_3$ (**a-c**), $Na_3V_2(PO_4)_3$ (**d-f**), $Na_{0.7}Fe_{0.7}Mn_{0.3}O_2$ (**g-i**), and $LiNi_{1/3}Co_{1/3}Mn_{1/3}O_2$ (**j-l**), and binary-metal oxides $LiMn_2O_4$ (**m-o**), $LiCoO_2$ (**p-r**), $NiCo_2O_4$ (**s-u**), and LiV_3O_8 (**v-x**) mesoporous nanotubes. The scale bar in **b**, **e** and **h** is 500 nm, the rest is 100 nm.

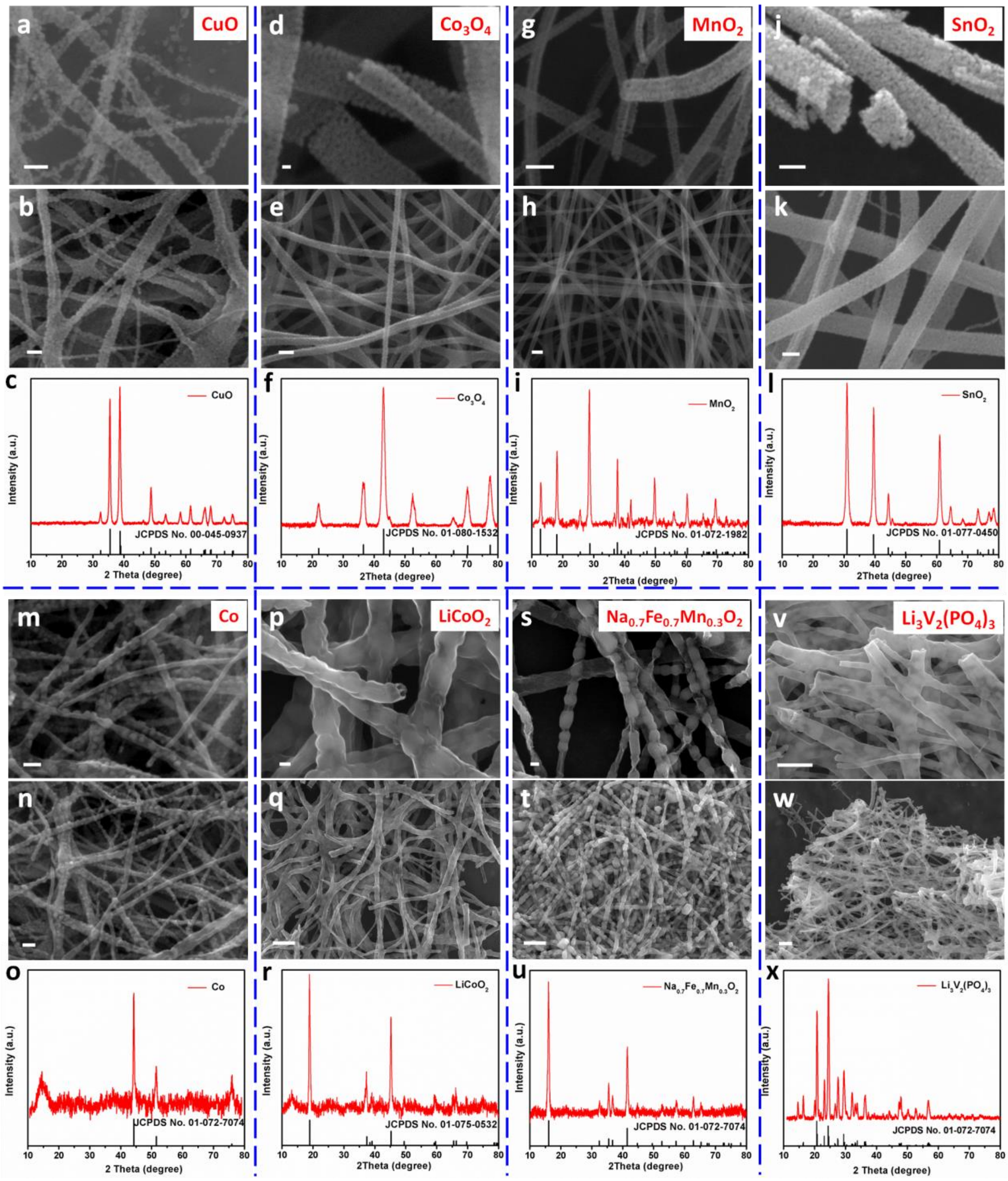

Supplementary Figure 3 | SEM images and XRD patterns of single-metal oxides mesoporous nanotubes, CuO (**a-c**), Co_3O_4 (**d-f**), MnO_2 (**g-i**), and SnO_2 (**j-l**), and pea-like nanotubes which are selected from different species, Co (**m-o**), $LiCoO_2$ (**p-r**), $Na_{0.7}Fe_{0.7}Mn_{0.3}O_2$ (**s-u**) and $Li_3V_2(PO_4)_3$ (**v-x**). The scale bar in **q**, **t, v** and **w** is 1 μm, the rest is 100 nm.

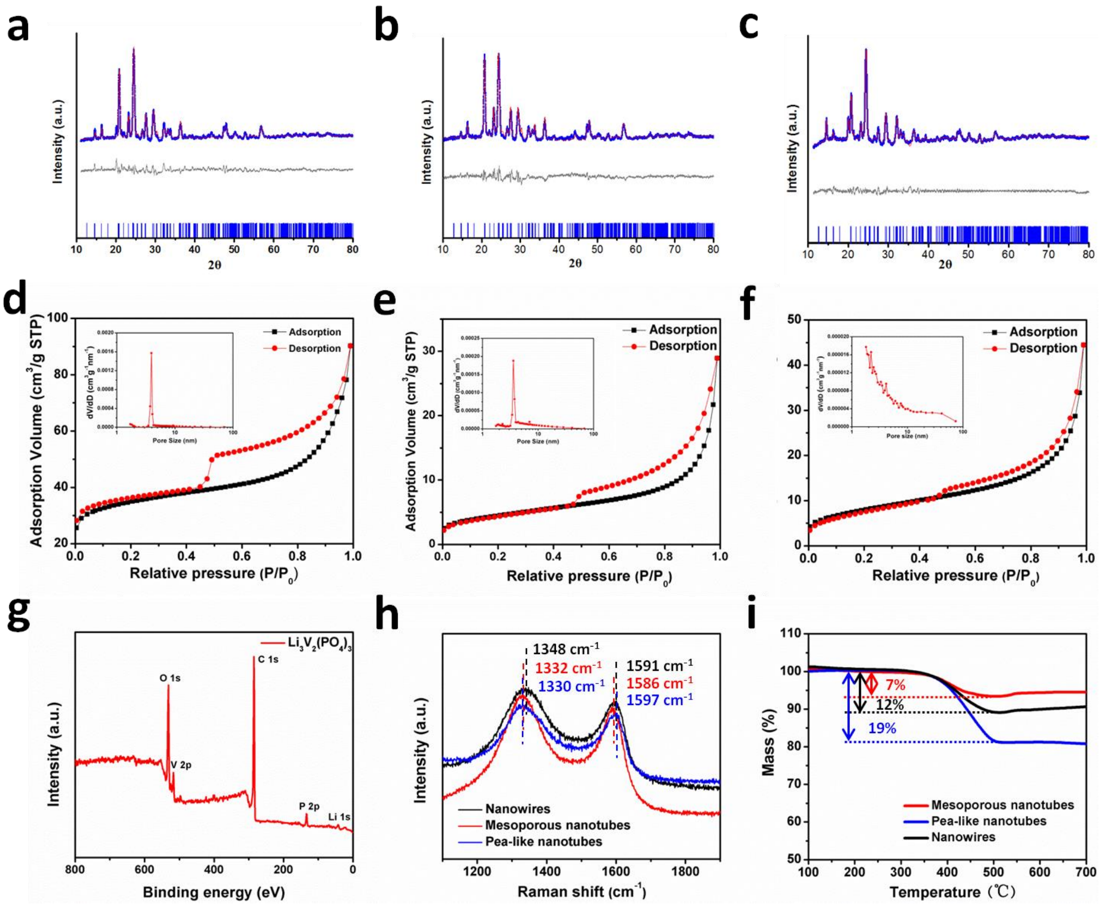

Supplementary Figure 4 | a-c, Profile fitting plots of $Li_3V_2(PO_4)_3$ mesoporous nanotubes (**a**), pea-like nanotubes (**b**) and nanowires (**c**). The crystal sizes of the $Li_3V_2(PO_4)_3$ mesoporous nanotubes, pea-like nanotubes and nanowires are 33.8, 106.8 and 22.3 nm, respectively, according to Rietveld analysis results; these sizes are consistent with those observed in SEM and TEM images. The XRD patterns are indexed to a monoclinic structure (JCPDS No. 01-072-7074). **d-f**, N_2 adsorption-desorption isotherms and pore size distribution curves (inset) for $Li_3V_2(PO_4)_3$ mesoporous nanotubes (**d**), pea-like nanotubes (**e**) and nanowires (**f**). The specific areas of these samples are 115, 33 and 15 m^2/g, respectively. **g**, X-ray photoelectron spectrum (XPS) of the $Li_3V_2(PO_4)_3$ mesoporous nanotube surface. **h-i**, Raman spectra (**h**) and TG curves (**i**) of $Li_3V_2(PO_4)_3$ mesoporous nanotubes, pea-like nanotubes and nanowires.

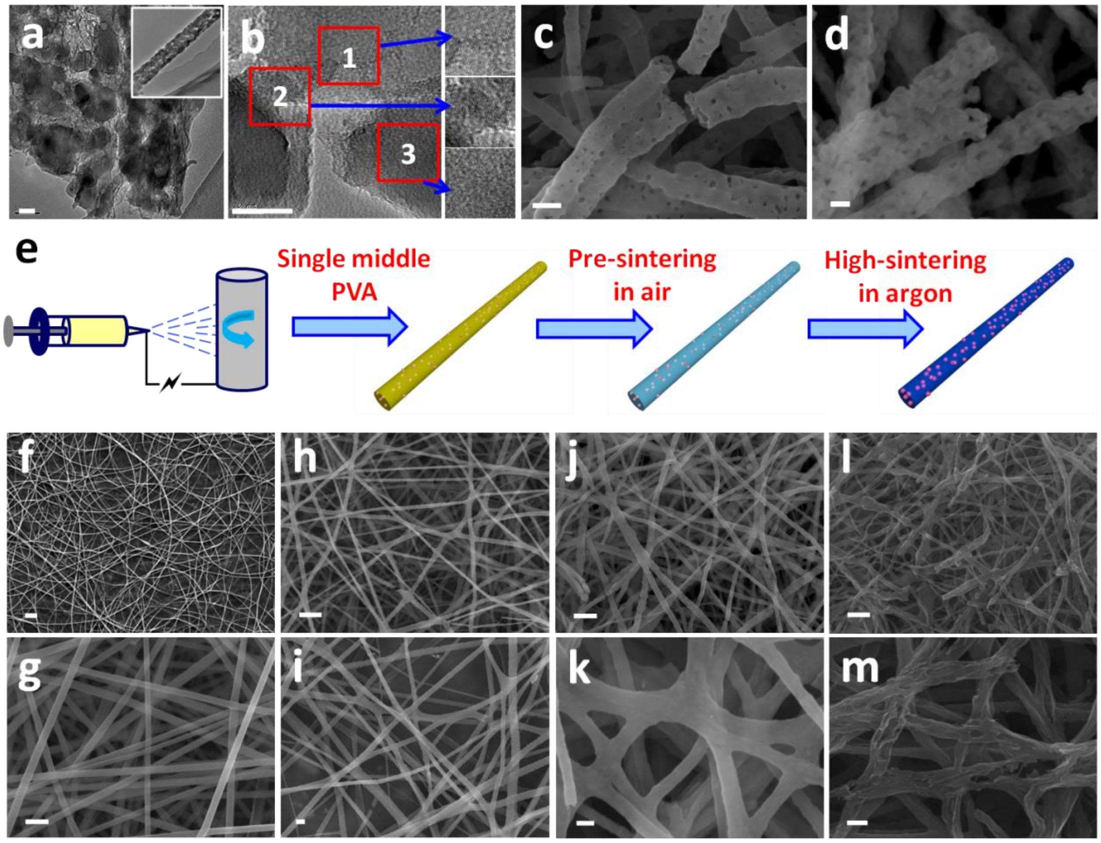

Supplementary Figure 5 | a-b, TEM (**a**) and high-resolution TEM (**b**) images of $Li_3V_2(PO_4)_3$ mesoporous nanotubes with scale bar at 20 nm. The inset of (**a**) is TEM image of one $Li_3V_2(PO_4)_3$ mesoporous nanotube. **c**, SEM image of $Li_3V_2(PO_4)_3$ mesoporous nanotubes pre-sintered at 300 °C for 10 h in air with scale bar at 100 nm. **d**, SEM images of $Li_3V_2(PO_4)_3$ mesoporous nanotubes pre-sintered at 350 °C for 10 h in air to remove additional PVA, getting larger pores on the nanotubes. The scale bar is 100 nm. **e**, Schematic diagram of electrospinning composite nanowires with only middle-molecular-weight PVA. **f-g**, SEM images of composite nanowires after electrospinning. **h-i**, SEM images of composite nanowires pre-sintered at 300 °C for 3 h in air. **j-k**, SEM images of $Li_3V_2(PO_4)_3$ nanowires subjected to high-temperature sintering at 800 °C for 3 h under argon. **l-m**, SEM images of carbon nanowires after removal of $Li_3V_2(PO_4)_3$ with hydrogen fluoride. The scale bar is 1μm in **f**, **h**, **j**, **l** and 200 nm in **g**, **i**, **k**, **m**. As a control sample, $Li_3V_2(PO_4)_3$ nanowires were also electrospun with only middle-molecular-weight PVA. First, the uniform precursor solution for electrospinning was prepared with 1.6 mol of middle-molecular-weight PVA and 20 ml of deionized water. Then, LiOH, NH_4VO_3 and $NH_4H_2PO_4$ were added in a molar ratio of 3:2:3. After being electrospun, the composite nanowires were annealed at 300 °C in air for 3 h, leading to pyrolysis of the middle-weight PVA. They were subsequently annealed at 800 °C under an argon atmosphere for 6 h.

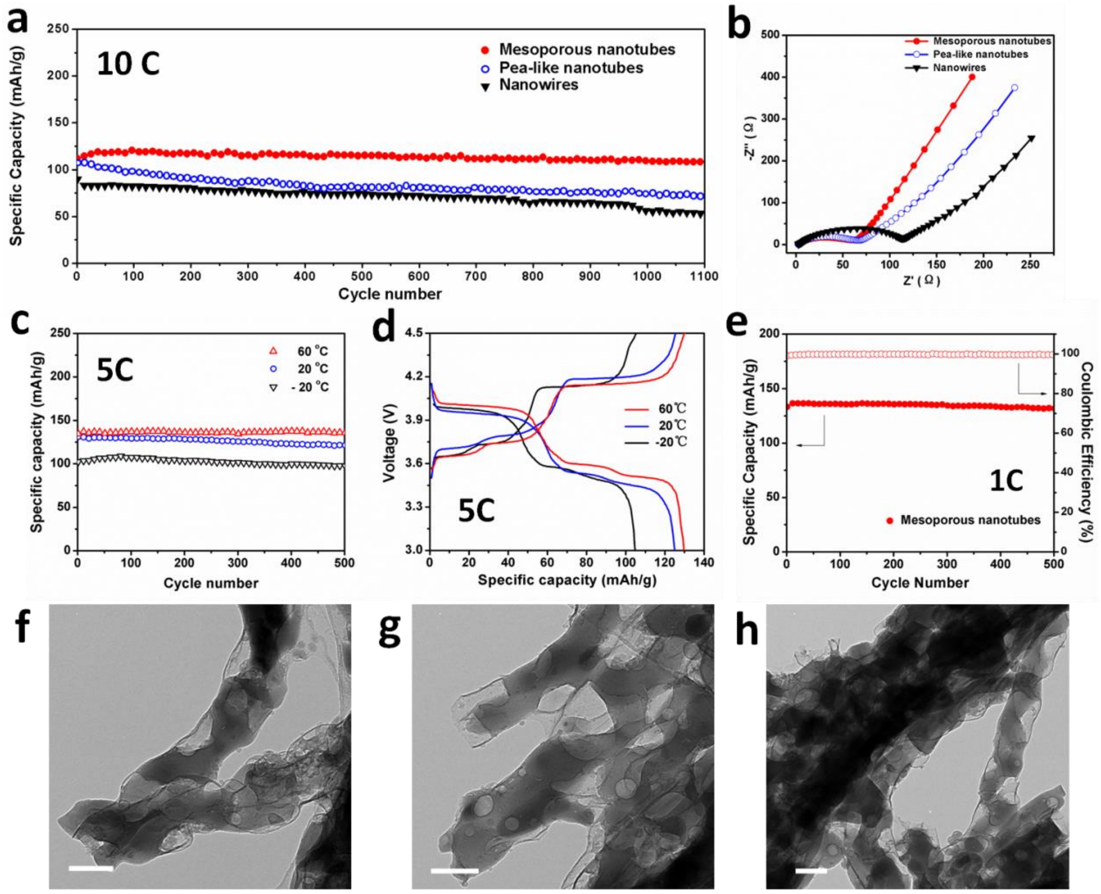

Supplementary Figure 6 | Electrochemical performance of $Li_3V_2(PO_4)_3$ nanowires, mesoporous nanotubes and pea-like nanotubes in lithium half-cells. a, Cycling performance of the half-cells measured at 10 C. **b**, AC impedance plots of these three samples. **c-d**, Cycling performance and charge-discharge curves collected at a rate of 5 C at temperatures of -20, 20 and 60 °C, respectively. **e**, Cycling performance and the corresponding coulombic efficiency measured at 1 C for 500 cycles. **f-h**, TEM images of $Li_3V_2(PO_4)_3$ mesoporous nanotubes after cycling 5000 times at the rate of 10 C with scale bar at 100 nm. At the high current density of 10 C, $Li_3V_2(PO_4)_3$ nanowires, mesoporous nanotubes and pea-like nanotubes deliver initial discharge capacities of 112, 107 and 90 mAh/g, respectively, after 1,100 cycles, the corresponding capacity retentions were 99%, 71% and 68%, respectively.

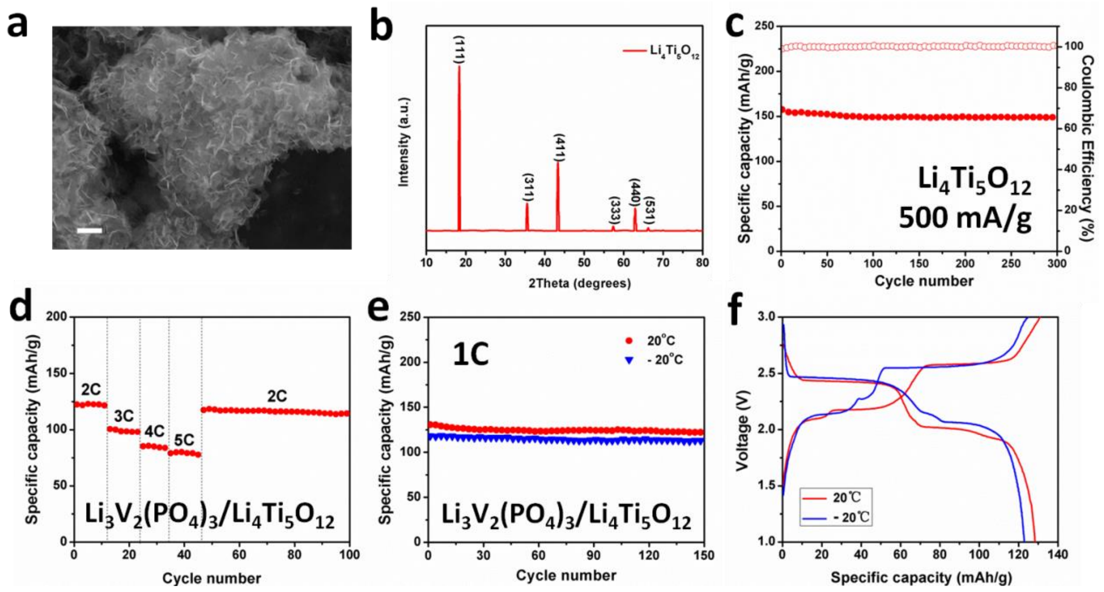

Supplementary Figure 7 | Electrochemical performance of $Li_3V_2(PO_4)_3/Li_4Ti_5O_{12}$ full batteries. a-b, SEM image (**a**) of $Li_4Ti_5O_{12}$ nanosheets with scale bar at 1μm, and the corresponding XRD pattern (**b**). **c**, Cycling performance of $Li_4Ti_5O_{12}$ nanosheets measured at 500 mA/g in a lithium half-cell. **d**, Specific capacity measurements at 20 °C of the $Li_3V_2(PO_4)_3/Li_4Ti_5O_{12}$ full battery at different rates ranging from 2 to 5 C. **e-f**, Cycling performance (**e**) and charge/discharge curves (**f**) of the $Li_3V_2(PO_4)_3/Li_4Ti_5O_{12}$ full batteries measured at a rate of 1 C and at temperatures of -20 °C and 20 °C. $Li_4Ti_5O_{12}$ half cell exhibited stable cycling performance and coulombic efficiency when measured at a rate of 500 mA/g. The $Li_3V_2(PO_4)_3/Li_4Ti_5O_{12}$ lithium-ion full batteries exhibited the initial capacities of 130 and 118 mAh/g when tested at temperatures of 20 °C and -20 °C, respectively, showing stable cycling performance at both room and low temperature.

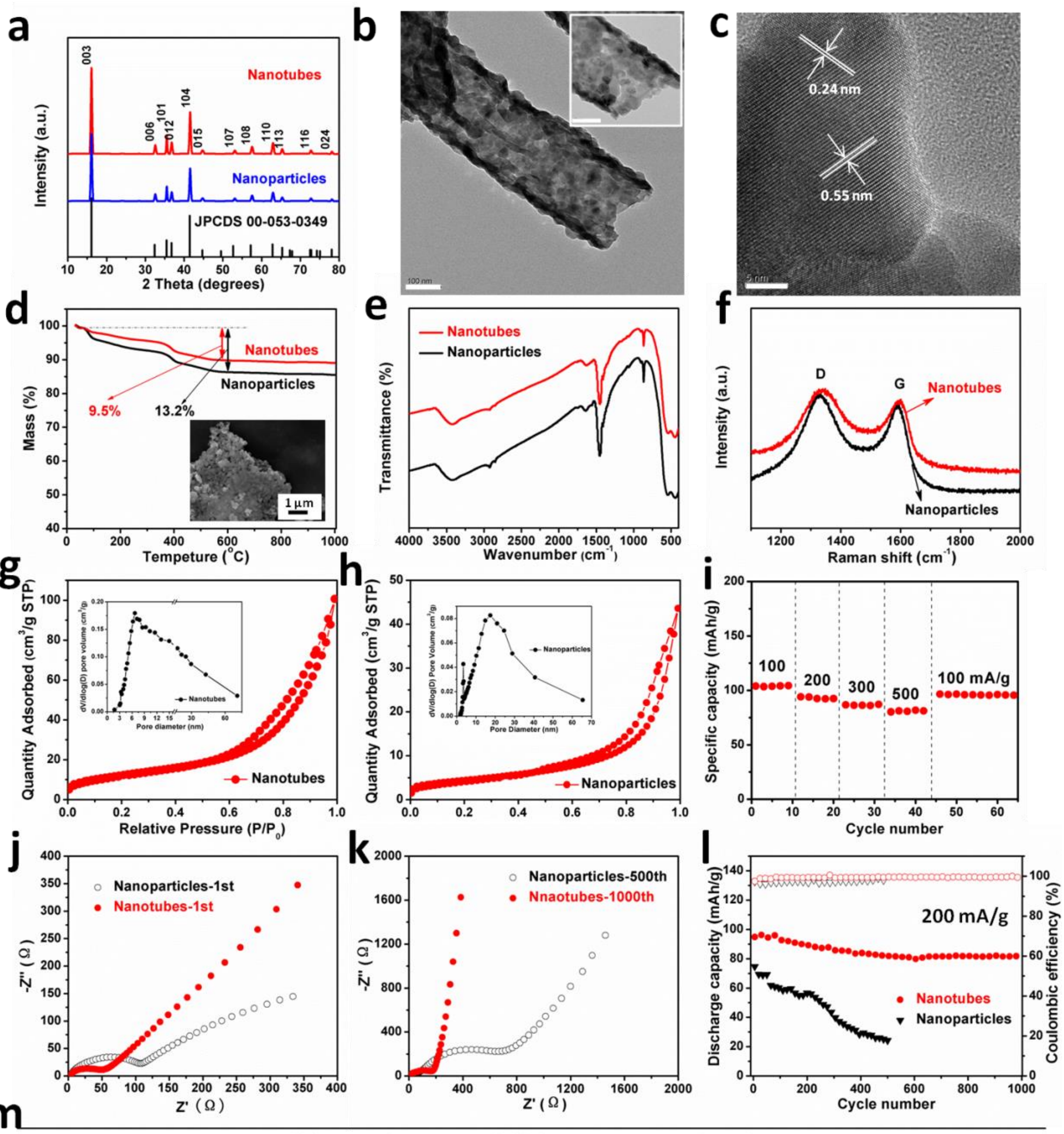

m

		Voltage range (V)	Current density (mA/g)	Initial Capacity (mAh/g)	Cycle number	Capacity fading per cycle	Reference
1	$Na_{0.7}Fe_{0.7}Mn_{0.3}O_2$	3.0-4.5	100	108	1000	0.0105%	Our work
			500	82	5000	0.0071%	
2	P2-$Na_{2/3}Fe_{1/2}Mn_{1/2}O_2$	1.5-4.2	13	190	30	0.78%	*Nat. Mater.*, 11 (7), 512 (2012)
3	P2-$Na_{2/3}Fe_{1/2}Mn_{1/2}O_2$	1.5-4.2	26	195	80	0.19%	*ACS Appl. Mater. Inter.*, 6, 8953 (2014).
4	P2-$Na_{1.0}Li_{0.2}Ni_{0.25}Mn_{0.75}O_2$	2.0-4.2	15	128	50	0.26%	*Chem. Mater.*, 26, 1260 (2014)
5	$Na[Ni_{0.25}Fe_{0.5}Mn_{0.25}]O_2$	2.1-3.9	130	133	50	0.30%	*Nano Lett.*, 14 (3), 1620 (2014)

Supplementary Figure 8 | Characterization and electrochemical performance of $Na_{0.7}Fe_{0.7}Mn_{0.3}O_2$ mesoporous nanotubes and nanoparticles in sodium-ion batteries. **a**, XRD patterns of $Na_{0.7}Fe_{0.7}Mn_{0.3}O_2$ mesoporous nanotubes and nanoparticles. **b**, TEM images of $Na_{0.7}Fe_{0.7}Mn_{0.3}O_2$ mesoporous nanotubes with scale bar at 100 nm. **c**, High-resolution TEM image of $Na_{0.7}Fe_{0.7}Mn_{0.3}O_2$ mesoporous nanotubes with scale bar at 5 nm. **d-f**, TG curves (**d**), FT-IR spectra (**e**) and Raman spectra (**f**) of the materials. **g-h**, N_2 adsorption-desorption isotherms and

pore size distribution curves (inset) of $Na_{0.7}Fe_{0.7}Mn_{0.3}O_2$ mesoporous nanotubes (**g**) and nanoparticles (**h**). **i**, Rate performance tested at 100, 200, 300 and 500 mA/g. **j-k**, AC impedance plots at the first cycle (**j**), 500th cycle and 1000th cycle (**k**). **l**, Cycling performance at 200 mA/g. **m**, A comparison of the electrochemical properties of the $Na_{0.7}Fe_{0.7}Mn_{0.3}O_2$ prepared in this work with those of the materials reported in the literatures. The HRTEM image displays lattice fringes with *d*-spacings of 0.55 and 0.24 nm, which correspond to the (003) and (012) planes, respectively, of the rhombohedral $Na_{0.7}Fe_{0.7}Mn_{0.3}O_2$ crystal. At the same time, compared with $Na_{0.7}Fe_{0.7}Mn_{0.3}O_2$ nanoparticles, which were synthesized as a control sample, $Na_{0.7}Fe_{0.7}Mn_{0.3}O_2$ mesoporous nanotubes exhibit much better electrochemical performance. The carbon mass content of $Na_{0.7}Fe_{0.7}Mn_{0.3}O_2$ mesoporous nanotubes is only 9.5%, which is smaller than nanoparticles (13.2%), but mesoporous nanotubes reveal larger specific surface area (95 m^2/g) than nanoparticles (15 m^2/g), which can greatly enhance the electrode-electrolyte contact area.

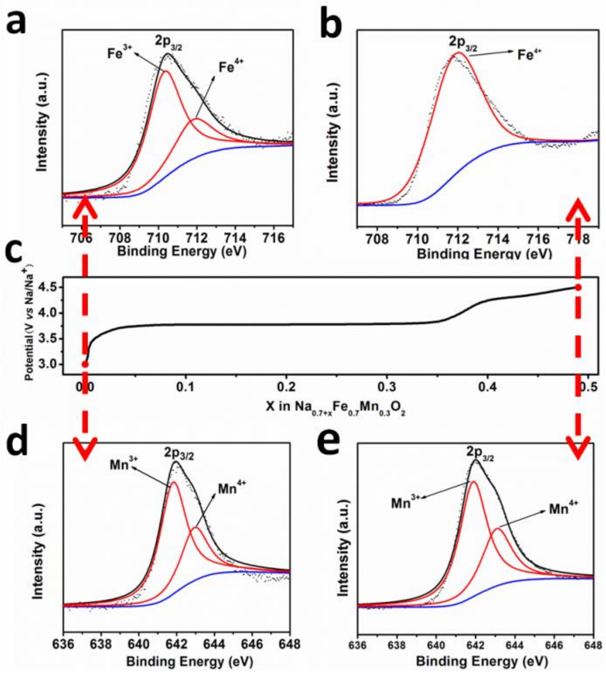

Supplementary Figure 9 | a-b, X-ray photoelectron spectra and Fe $2p_{3/2}$ curve fits for the original $Na_{0.7}Fe_{0.7}Mn_{0.3}O_2$ mesoporous nanotubes surface (**a**) and for the material charged to 4.5 V (**b**). **c**, Evolution of the cell voltage as a function of the sodium content in $Na_{0.7+x}Fe_{0.7}Mn_{0.3}O_2$ over the $0 \leq x \leq 0.49$ composition range. **d-e**, X-ray photoelectron spectra and Mn $2p_{3/2}$ curve fits for the original $Na_{0.7}Fe_{0.7}Mn_{0.3}O_2$ mesoporous nanotube surface (**d**) and for the material charged to 4.5 V (**e**). This measurement clearly demonstrates that the discharge capacity of $Na_{0.7}Fe_{0.7}Mn_{0.3}O_2$ mesoporous nanotubes mainly comes from the contribution of iron element.

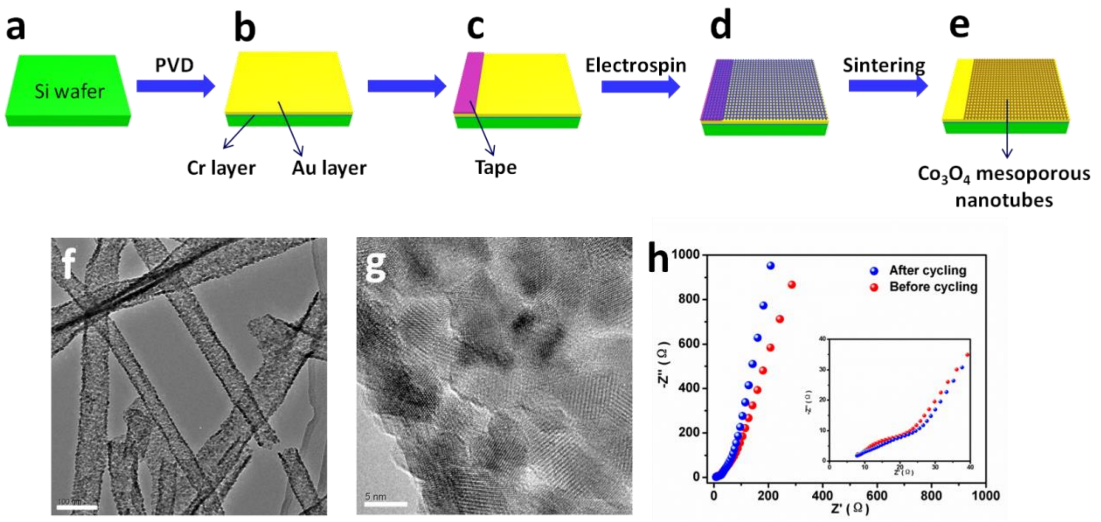

Supplementary Figure 10 | a-e, Schematic of the fabrication process for a Co_3O_4 mesoporous nanotube based device integrated onto a silicon wafer. **a**, The cleaned silicon wafer. **b**, The chromium layer (~5 nm) and gold layer (~50 nm) deposited on the silicon wafer using physical vapor deposition (PVD). **c**, Tape placed on the surface of one side. **d**, Electrospinning of Co_3O_4 onto the surface. **e**, Tape removal and sintering at 350 °C in air for 5 h to acquire a layer of Co_3O_4 mesoporous nanotubes on the surface. The thickness of the Co_3O_4 mesoporous nanotubes was ~850 nm. **f-g**, TEM images of the resulting Co_3O_4 mesoporous nanotubes. **h**, AC impedance plots of the Co_3O_4 mesoporous nanotubes based supercapacitor. The inset is a magnified plot of the high-frequency region.
